# Patients’ view of routine follow-up after breast cancer treatment

**DOI:** 10.1007/s00508-017-1278-8

**Published:** 2017-10-17

**Authors:** Vesna Bjelic-Radisic, Martha Dorfer, Karl Tamussino, Elfriede Greimel

**Affiliations:** Department of Obstetrics and Gynecology, Medical University Graz, Auenbruggerplatz 14, 8036 Graz, Austria

**Keywords:** BC-PASS, Breast cancer, Follow-up, Patient-reported outcome, Aftercare

## Abstract

**Background:**

To assess emotional distress, anxiety and stress reactions in breast cancer (BC) patients before the follow-up visits.

**Study design:**

Between September 2009 and December 2011 a total of 284 patients completed the BC-psychosocial assessment screening scale (PASS) and a questionnaire about their views of follow-up after treatment for BC.

**Results:**

Of the patients 64% reported low level of distress on the BC-PASS. The mean scores on the physical well-being scale was 5.3, the satisfaction/sense of coherence scale 7.4, and the emotional distress scale 8.1. Women rated mammography as the most important component (71%), followed by breast ultrasound (63%) and the consultation with the physician (60%). Of the patients 94% were satisfied with the current follow-up regimen.

**Conclusions:**

In this series BC patients were satisfied with their aftercare. Mammography was thought to be the most important component of aftercare. Patient-reported outcomes should be taken into account when planning follow-up.

## Introduction

Follow-up of women treated for breast cancer includes evaluation of any ongoing treatment, side effects, evaluation of disease status, detection of recurrence, and providing information and psychosocial support [1–3]. Most clinical guidelines for follow-up of patients with breast cancer recommend follow-up every 3–6 months in the first 2 years, every 6 months in the following 3 years, and annually thereafter [2–5]. Follow-up should include taking a medical history, physical examination and annual mammography [2–5]. There is a lack of evidence that more intensive follow-up (including for example chest x‑ray, bone and liver scans, serum tumor markers, blood and liver tests) are better at detecting recurrence than periodic physical examination and annual mammography [1–7]. While there is no evidence that more intensive follow-up improves survival [1, 5–7], patients desire follow-up care and surveillance to reduce their fear of recurrence and for psychosocial support [8–15]; however, continuity of care and psychosocial support are very important for women with breast cancer and such patient-centered aspects should be taken into account when considering changes in follow-up plans [12–16]. Follow-up appointments are associated with anxiety, with a peak 2 days before the appointment, particularly with respect to the possibility of recurrence [11, 17–19].

The present study assessed emotional distress, anxiety and stress reactions in breast cancer patients before routine follow-up visits. We also surveyed patients views regarding their follow-up schedule.

## Patients and methods

### Setting

Women were recruited at our Breast Unit during regularly scheduled follow-up visits between September 2009 and December 2011 after completion of locoregional therapy and/or chemotherapy for invasive breast cancer (IBC) or ductal carcinoma in situ (DCIS). Inclusion criteria were a history of breast cancer diagnosed at least 3 months previously with completed locoregional therapy and/or chemotherapy, willingness to participate in the study, and sufficient German language skills. Clinical data included the TNM stage of disease at diagnosis, treatment modalities, time since diagnosis, and disease status as well as treatment modalities at the time of assessment (Table 1).

**Table 1:** Clinical and demographic characteristics of study sample (*N* = 284), Age (median) 59.5 years (SD±11.9 years)

Tumor stage	*N*	(%)
Non-invasive carcinoma	24	8.7
*Invasive carcinoma*		
T1	176	61.9
T2	63	22.1
T3	9	3.0
T4	8	3.0
Unknown	4	1.3
*Nodal status*		
N0	187	65.8
≥N1	93	32.7
Unknown	4	1.5
Metastases/recurrence	29	10.4
*Treatment*		
*Surgery*		
BET	224	78.9
Mastectomy	55	19.4
No surgery	5	1.7
SN biopsy	128	45.2
Axillary lymph node dissection	117	41.5
*Chemotherapy*		
Neoadjuvant	24	8.4
Adjuvant	122	42.8
Irradiation	233	81.9
Hormonal therapy	218	76.9
*Relationship*		
Single	16	5.7
Married/living with partner	172	60.5
Divorced/widowed	79	27.8
Unknown	17	6
*Education*		
Compulsory	121	42.2
Post-compulsory	149	52.8
University level	14	5.0
*Employment*		
Employed	77	27.1
Retired	86	30.3
Homemaker/unemployed	88	31.0
Unknown	33	11.6

*BET* Breast conserving Therapy, *SN* SN Biopsy

All patients were asked to complete the breast cancer psychosocial assessment screening scale (BC-PASS) and a questionnaire about their views of follow-up [20]. A trained research assistant interviewed patients without clinical staff present in order to minimize response bias. Clinical data included TNM stage of disease at diagnosis, treatment modalities, time since diagnosis, disease status as well as treatment modalities at the time of assessment (Table 1).

Follow-up consisted of taking a medical history and physical examination at 3‑month intervals during the first 2 years after primary treatment, at 6‑month intervals between years 3 and 5, and at yearly intervals thereafter. Follow-up visits were at the hospital unit. Patients in follow-up with long-term therapy, such as hormonal therapy were included in this study. Mammography and breast ultrasound were scheduled at yearly intervals. Liver ultrasound, chest x‑ray and tumor markers were obtained at yearly intervals until 2010. Laboratory testing was at the discretion of the attending physician. Further tests were ordered as indicated. At 5 years patients chose whether to continue follow-up at the breast unit or with a physician of choice.

### Follow-up survey

We reviewed the literature on views of patients and physicians regarding follow-up models to identify areas that patients found important. Based on these areas, we developed a follow-up questionnaire in a multidisciplinary team of physicians, clinical psychologists, gynecologist/breast cancer specialists and patients. The survey was pilot tested in face-to-face interview in a sample of 20 patients.

The follow-up questionnaire included the following domains:

1. Sociodemographic data: age, marital status, educational level, employment status.
2. View of follow-up regarding frequency of appointments.
3. Satisfaction with follow-up visit (4-point Likert scale, 1 = not at all, 4 = very much).
4. Importance and distress regarding the components of follow-up, e.g. consultation with physician, physical (breast) examination, mammography, breast ultrasound, gynecological examination, abdominal ultrasound, chest x‑ray, serum tumor markers, computed tomography (CT), magnetic resonance (MR) mammography, bone densitometry, positron emission tomography or other examinations if performed, on a 4-point Likert scale (1 = not at all to 4 = very much).
5. Services available throughout the follow-up period: psychological support, rehabilitation and social services, self-help groups and nutritional support.
6. Emotional status before follow-up visits: anxiety level on a 10-point Likert scale (0 = lowest and 10 = highest level) and stress symptoms (irritability/tension distressing thoughts/worries, fear of recurrence) on a 4-point Likert scale (1 = not at all to 4 = very much).

### Psychological assessment screening scale

The BC-PASS was used to assess psychological distress [20]. The BC-PASS addresses three domains:

A. physical well-being (2 items),
B. satisfaction/sense of coherence (3 items) and
C. emotional distress (3 items).

The item responses comprised scores from 1–7 on a Likert scale. For each factor a summary score and cut-off was established: for physical well-being a score ≥9 indicated a high level of physical impairment, for satisfaction and emotional distress a score ≥14 indicated a high level of emotional stress.

### Statistical analysis

Sociodemographic and clinical data were analyzed using descriptive statistics. Data were analyzed using Statistical Package for the Social Sciences (SPSS) version 16.0 [21].

## Results

A total of 299 patients were invited to participate in the study and 15 women (5%) refused participation or did not return the questionnaire so that the study was based on 284 patients (median age 59 years, range 29–86 years). Clinical and demographic data are shown in Table 1. The majority of patients were married (61%) and 69% had at least compulsory school education. There were no differences between responders and non-responders to the study assessment in clinical or demographic characteristics. The median follow-up was 41 ± 52 months after primary treatment, 148 (52%) patients were in the first 2 years of follow-up, 88 (31%) patients were in years 2–5 and 48 (17%) had completed treatment more than 5 years ago. Of the patients 29 (10.4%) developed a recurrence during the follow-up period (Table 1).

The large majority of patients (87%) considered the current frequency of the follow-up visits appropriate: 12 (4%) wanted more frequent appointments, 18 (6%) felt they were being seen too often and 3 (1%) desired an appointment only when they experienced symptoms. On a 4-point Likert scale 94% of patients were very satisfied or satisfied with the current follow-up regimen. Only 6 patients (2%) were very dissatisfied, 15 women (5%) did not like the presence of more than 1 person during the examination and 77% of patients were very satisfied with the organization and setting in the clinic. Patients considered mammography the most important component of the follow-up schema (71%), followed by breast ultrasound (63%) and the consultation with the physician (60%) (Table 2).

**Table 2:** Importance of different follow-up examinations (*N* = 284)

Examination	High level of importance		Low level of importance	
	*N*	%	*N*	%
Consultation with physician	171	60	113	40
Breast palpation	157	55	122	43
Mammography	203	71	76	27
Breast ultrasound	178	63	101	35
Gynecological examination	136	48	148	52
Abdominal ultrasound	92	32	192	68
Chest x‑ray	106	37	178	63
Blood test with tumor markers	120	42	164	58
Imaging (CT, MRI,PET)	96	34	186	65
Bone scintigraphy	104	37	178	63

*CT* computed tomography, *MRI* magnetic resonance imaging, *PET* positron emission tomography

Answer with high or low importance are shown in the table,only.

Overall, on the 4‑point Likert scale 14% of patients considered mammography distressing and only 1% considered the consultation itself distressing (Table 3).

**Table 3:** Level of distress experienced in the follow-up (*N* = 284)

	None		Low		Moderate		High	
	*N*	%	*N*	%	*N*	%	*N*	%
Consultation with physician	214	72	42	14	10	3	3	1
Breast palpation	173	58	64	21	22	7	5	2
Mammography	89	30	68	23	63	21	41	14
Breast ultrasound	145	49	64	21	28	9	11	4
Gynecological examination	95	32	95	32	42	14	19	6
Abdominal ultrasound	141	47	69	23	22	7	6	2
Chest x‑ray	147	49	60	20	25	8	8	3
Blood test with tumor markers	101	47	66	22	31	10	16	5
Imaging (CT, MRI,PET)	98	33	53	18	41	14	13	4
Bone scintigraphy	110	37	62	21	34	11	12	4
*Distress symptoms*								
Irritable before the visit	105	35	86	29	53	18	19	6
Fear of recurrence	76	25	85	28	76	25	24	8
Worry before the visit	109	37	75	25	48	16	22	7

*CT* computed tomography, *MRI* magnetic resonance imaging, *PET* positron emission tomography

Regarding distress symptoms 24% of patients reported themselves “to be moderate to highly irritable” prior to the visit. Approximately one half of the patients reported low levels of fear of recurrence (54%) and 61% were “not at all” or “a slightly” worried several days before the visit (Table 3). Of the patients 64% reported low levels of anxiety prior to the follow-up visit (score ≤3 on 10-point Likert scale), 26% of patients reported moderate anxiety (score ≥4–6) and 6% had severe anxiety (score ≥7). The questionnaire aimed to identify the topics that patients wished to be addressed during a follow-up visit. Of the patients 37% desired more information about the etiology of breast cancer, 54% were interested in improving their immunologic function, 30% wanted information about nutrition, 30% about rehabilitation services and 17% of patients indicated that information about psychological support was inadequate. Using the BC-PASS the majority of patients reported a low level of distress (Table 4). The mean score for factor A (physical well-being scale) was 5.3 (SD 3.1), for factor B (satisfaction/sense of coherence) 7.4 (SD 4.1) and for factor C (emotional distress scale) 8.1 (SD 4.2). Of the patients 14.6% had a cut-off score ≥9 indicating high levels of physical impairment, 6.6% of patients on the satisfaction/sense of coherence scale and 10% on the emotional distress scale had cut-off scores ≥14 indicating a high level of emotional stress symptoms (Table 4).

**Table 4:** Mean scores of the psychological assessment screening scale

Factor	Mean	SD	Cut-off score^a^	%
A: Physical well-being	5.3	3.1	≥9	14.6
B: Satisfaction/sense of coherence	7.4	4.1	≥14	6.6
C: Emotional distress	8.1	4.2	≥14	10.4

^a^Higher score indicates a higher level of problems.

## Discussion

Our study sample with a median age of 59 years is representative for breast cancer patients. The majority of women attending routine follow-up visits after breast cancer at our unit were satisfied with their follow-up schema, 87% of patients considered the frequency of follow-up visits appropriate although follow-up visits were frequent, especially during the first 2 years. Our results are in lines with published data. Lewis et al. reported that the main reason for willingness to have regular surveillance is patients’ anxiety and fear of recurrence, especially during the early phase after completing treatment [15]. Continuity of care and consultation were most important for patients and patients valued the expertise of specialists in hospital settings [15]. Psychological distress, anxiety and depression are common among breast cancer patients even years after diagnosis and therapy [22]. It has long been known that very frequent visits with annual mammography and physical examination do not improve detection rates of recurrence [23]. More frequent follow-up does not appear to prolong overall survival [1, 5, 24]. The MaCare trial found that less intensive and less frequent follow-up did not impair quality of life (QoL) or prolong the time to detection of recurrences and concluded that the most important component is psychosocial support and information [4]. Although lower frequency of follow-up is associated with poorer survival due to delayed detection of recurrence, patients views and needs for psychological support are important. One model that covers both points is education of patients regarding goals and effectiveness of follow-up with guaranteed access to health specialists in case of questions and problems [25]. Only 13% of patients were not satisfied with the schedule of follow-up visit. In a randomized trial Grunfeld et al. reported similar results with 7% of patients refusing to participate in the study because of reduced frequency of follow-up [25]. Some patients in our study preferred longer intervals between visits, for others anxiety persisted or increased with lengthened intervals [7, 8, 10]. For such patients personalized follow-up according to preference and risk stratification is a good option [16].

In our study patients considered mammography the most important single component of follow-up, followed by breast ultrasound and the consultation itself. Patients did not consider more intensive imaging techniques to be important. These results are in line with our current follow-up schedule and clinical guidelines [2, 3]. Patients in our study wanted more information on the etiology of breast cancer, improving their immune function, nutrition and rehabilitation services. Thus, feedback from patients is necessary to identify areas for improvement based on patient needs. The patient satisfaction with current follow-up schedule still continues to be an important factor for healthcare quality [26]. Contrary to data that patients with cancer are at high risk of emotional disorders including anxiety, traumatic stress and depression [27, 28], our study patients reported low levels of anxiety. More than 50% of our patients had little fear of recurrence. We could not confirm that in our setting fear of recurrence is the key factor for the desire to have regular follow-up visits as reported previously [9].

Regarding distress symptoms 24% of patients reported “to be moderately to highly irritable” prior to the visit. The distress can be intensified by long waiting times, lack of information, poor communication with the staff, and the absence of psychosocial care [29]. These results enable identification and recognition of patient problems and the possibility to improve our strategies [26]. Of our patients 77% were satisfied with the organization and setting of the outpatient clinic and highly satisfied with their follow-up schedule. Patients showed a strong preference for the existing regime. This finding has to be included in evaluating the quality of care, despite growing costs of routine follow-up.

The strengths of the study are a psychological assessment with a validated instrument as well as a follow-up survey developed in consultation with various specialists and patients. Limitations of the study are a potential selection bias as we only asked patients attending follow-up and the wide diversity of patients. Patient-reported outcomes should be taken into account when considering changes in the provision of follow-up care for breast cancer patients. Feedback from patients is necessary to identify areas for improvement based on patient needs.

## Conclusion

Breast cancer patients rated mammography as the most important component of the follow-up visit. Patient-reported outcome should be taken into account when considering changes in the provision of follow-up care.
